# Malaria Infection and Anemia Prevalence in Zambia's Luangwa District: An Area of Near-Universal Insecticide-Treated Mosquito Net Coverage

**DOI:** 10.4269/ajtmh.2011.10-0287

**Published:** 2011-01-05

**Authors:** Thomas P. Eisele, John M. Miller, Hawela B. Moonga, Busiku Hamainza, Paul Hutchinson, Joseph Keating

**Affiliations:** Department of International Health and Development, Tulane University School of Public Health and Tropical Medicine, New Orleans, Louisiana; Partnership for Appropriate Technology (PATH) in Health Malaria Control and Evaluation Partnership in Africa (MACEPA), Chainama Hospital, Lusaka, Zambia; National Malaria Control Centre, Ministry of Health Zambia, Chainama Hospital, Lusaka, Zambia

## Abstract

We examined the relationship between insecticide-treated mosquito nets (ITNs), malaria parasite infection, and severe anemia prevalence in children in Luangwa District, Zambia, an area with near-universal ITN coverage, at the end of the 2008 and 2010 malaria transmission seasons. Malaria parasite infection prevalence among children < 5 years old was 9.7% (95% confidence interval [CI] = 8.0–11.4%) over both survey years. Prevalence of severe anemia among children 6–59 months old was 6.9% (95% CI = 5.4–8.5%) over both survey years. Within this context of near-universal ITN coverage, we were unable to detect a significant association between malaria parasite or severe anemia prevalence and ITNs (possession and use). In addition to maintaining universal ITN coverage, it will be essential for the malaria control program to achieve high ITN use and laboratory diagnosis and treatment of all fevers among all age groups to further reduce the malaria burden in this area.

## Introduction

Community randomized controlled trials have shown insecticide-treated mosquito nets (ITNs) to be an effective strategy for preventing malaria morbidity and all causes of mortality among children in *Plasmodium falciparum* endemic settings.^1^ These trials show ITNs to have a pooled protective efficacy of reducing the prevalence of malaria parasite infection in children by 17% within areas of stable transmission.^2^ Trials have also shown that children protected by ITNs have a reduced risk of anemia, with a 1.7% absolute higher-packed cell density than unprotected children.^1^

There has recently been a call for universal coverage of ITNs across malaria endemic countries in Africa, defined in the Global Malaria Action Plan as 100% coverage of ITNs among at-risk populations.^3^ There are now household surveys in many malaria endemic countries of Africa, which include biomarkers for measuring the prevalence of malaria parasite infections and severe anemia in children, that can be used for monitoring and evaluating malaria control programs, especially within countries scaling-up ITN coverage. However, to date, there are relatively little data showing the relationship between ITNs (household possession and use) and malaria parasite infection prevalence in children under program conditions that have achieved near-universal coverage. Because of the community effect observed during trials, where ITNs confer protective benefits to unprotected children at very high intervention coverage levels,^4^,^5^ reductions in malaria parasite prevalence associated with ITNs may be lower than expected as near-universal coverage is achieved under program conditions.

This paper examines the relationship between ITN possession and use and the outcomes of malaria parasite infection and severe anemia in children in Luangwa District, Zambia, in 2008 and 2010, an area with near-universal coverage of ITNs. The objectives of this paper are to (1) report malaria parasite infection prevalence and severe anemia prevalence in children by ITN possession and use exposure and (2) determine whether ITN possession and use exposure are associated with reduced risk of malaria parasite infection or severe anemia among children within this context while controlling for potential confounding factors.

## Materials and Methods

### Study site description.

Luangwa district is located in Eastern Lusaka Province and is situated along the Luangwa River at the confluence with the Zambezi River. The entire district, with a total population of approximately 34,000 people, is classified as rural by the Zambian Central Statistics Office. The only town is Luangwa Boma, located at the southernmost end of the district. Villages are primarily situated along the Luangwa River, with fishing and small-scale agriculture being the primary livelihoods of the population. The district is served by nine rural health centers, two of which have both inpatient and outpatient services.

The district has a history of high malaria transmission, with a marked seasonal pattern after the rains from December to April. Luangwa district was one of the first districts in Zambia to be targeted for the scale-up of ITNs, serving as the pilot for mass distribution in 2005. Approximately 16,000 ITNs, of which 14,000 were long-lasting ITNs (LLINs), were distributed through mass free distribution and resale at heavily subsidized prices from 2005 to 2006 (LLINs and ITNs are henceforth referred to here as ITNs). As a result, there were enough ITNs to achieve a ratio of three nets per household in the district by the end of 2006. No indoor residual spraying had been done in Luangwa District. Rapid diagnostic tests (RDTs) have been used since 2006 throughout the public sector for malaria diagnosis, with artemisinin-combination therapy (ACT) (Coartem, Novartis, Basel, Switzerland) used as the first-line treatment of malaria since 2004.

### Survey methods.

As described elsewhere,^6^ a baseline household survey was conducted at the end of the peak malaria transmission season in Luangwa in April–May 2008 to assess anemia and malaria parasite infection prevalence among children < 5 years old, malaria-related knowledge, attitudes and practices, and malaria intervention coverage. A follow-up survey using the same methods was done in April 2010. Both surveys used a simple random sample of households by a complete digital enumeration of all households in the district in March 2008 using global positioning system (GPS) technology. As part of a community randomized controlled trial being conducted in the district to assess a community-based intervention to increase the use of ITNs by children, a total sample size of 1,200 children across both surveys was sought to allow the detection of a 10% increase in ITN use from a baseline of 50% with 80% statistical power, assuming a design effect of 1.35 and the probability of committing a type-1 error set at 5% (one-tailed test). A secondary hypothesis in this study was to test if ITNs (household possession and use) are associated with parasite prevalence within this context of near-universal ITN coverage. The total sample size of 1,200 children is sufficient to detect an absolute decrease in malaria parasite prevalence of 2.9% from a baseline of 10% with greater than 80% statistical power, assuming the probability of committing a type-1 error is 5% (one-tailed test).

### Measurement of outcomes.

The Luangwa baseline survey followed the standardized malaria indicator survey (MIS) protocol that was used for the 2008 and 2010 Zambia National MIS. Blood slides were sought for all children < 5 years old within selected households. Malaria blood slides were read at the National Malaria Control Center, Ministry of Health, by two trained laboratory technicians using standard methods.^7^ Blood slides were considered negative if no parasites were found after counting 200 thick film fields at 1,000× magnification. Hemoglobin (Hb) levels within children were ascertained using HemoCue. The blood slides of 10 children could not be matched to a unique identification number and were excluded from the analysis. Malaria parasite infection prevalence is defined as the proportion of sampled children < 5 years old with any detectable parasites out of all children < 5 years old that provided a blood sample. Severe anemia is defined as the proportion of sampled children 6–59 months old with Hb levels < 8 g/dL out of all sampled children this age. Children with a malaria parasite infection and/or anemia were treated immediately as per standard of care in Zambia.

Standardized household and women's questionnaires were used to collect information in accordance with the MIS protocol (http://www.rollbackmalaria.org/toolbox/tool_MISToolkit.html). ITN possession and use among all household residents were ascertained with a net roster and a registry of all household residents. Exposure of a child to household protection by an ITN (ITN possession) is defined in the following two ways: (1) the proportion of sampled children living in a household with more than or equal to one ITN, and (2) the proportion of sampled children living in households with at least one ITN per two residents (e.g., ITN to occupant ratio), which provides ideal coverage for a household. Exposure of a child to personal protection by an ITN (e.g., ITN use) is defined in the following two ways: (1) the proportion of sampled children living in a household reported to have slept under an ITN the night before the survey, and (2) the proportion of children living in a household where any ITN was used by any household member the night before the survey. ITN possession analyses are among all children providing a blood sample (*N* = 1,190). ITN use analyses are among all children providing a blood sample as well as among children providing a blood sample living in households with greater than or equal to one ITN to assess the effect of personal protection independent of having access to an ITN in the household (*N* = 969).

Ethical approval was obtained from the Institutional Review Boards (IRB) of Tulane University, the University of Zambia, and the Partnership for Appropriate Technology in Health (PATH).

### Data analysis.

Stata 10.1 (Stata Corporation, College Station, TX) was used to perform all statistical analyses. Data from the 2008 and 2010 surveys were pooled after determining that the association between ITN exposure (household possession and use) and the primary outcomes were homogeneous across survey rounds. ArcGIS was used to divide Luangwa District into north (≥ 50 km from the southern border) versus south (< 50 km from the southern border); in this analysis, the distance division is based on the parasite prevalence described elsewhere and is intended to capture variation in ecological conditions,^6^ which could influence the intensity of malaria transmission and resultant effect of ITNs. A household wealth index was constructed based on a principal components analysis of household assets.^8^ χ^2^ statistics were used to test for differences in severe anemia and parasite prevalence by ITN possession and use variables and between anemia and parasite prevalence. Logistic regressions were used to test whether ITN possession and use predicted parasite prevalence in children < 5 years old or severe anemia among children 6–59 months while controlling for age and sex of the child, location in the district, and household wealth; a total of four regressions models were performed. Wald statistics were used to identify variable significance in the models, with the probability of committing a type-1 error (α) set at 0.05. Standard errors for all point estimates and regression parameters were empirically estimated to account for correlated data at the household level.

## Results

Of a total of 1,595 households sampled in 2008 and 2010, 914 households contained children < 5 years old (*N* = 483 in 2008; *N* = 431 in 2010). The total number of eligible children in these households was 1,440, of which consent from a parent or guardian for their child to provide a blood sample was received for 1,190 children, representing a non-response rate of 15.2%.

Among all children < 5 years old in the samples, 81.1% (95% confidence interval [CI] = 79.0–83.1%) slept in a house possessing ≥ 1 ITN. Among children < 5 years old that provided a blood sample for microscopy diagnosis, 81.4% (95% CI = 78.5–84.3%) slept in a house with ≥ 1 ITN (Table 1). ITN household possession increased with household wealth (χ^2^ = 24.8; *P* < 0.01). No other significant differences in ITN household possession were detected.

Among children providing a blood sample for microscopy diagnosis, 19.1% (95% CI = 16.8–21.8%) slept in a house with ≥ 1 ITN per two occupants (Table 1). The proportion of households with ≥ 1 ITN per two occupants generally increased with household wealth (χ^2^ = 44.9; *P* < 0.01); no other significant differences in intrahousehold access to ITNs were detected.

ITN use the previous night among children in ITN-owning households increased from just over one-half (52.8%; 95% CI = 48.4–57.2%) in 2008 to 85.5% (95% CI = 81.2–87.8%) in 2010. The combined proportion of children sleeping under an ITN the previous night was 68.0% (95% CI = 64.6–71.4) (Table 1). Among children in ITN-owning households, one-third (30.3%; 95% CI = 27.0–33.6%) lived in a house where none of the ITNs were used the night before the survey.

**Figure 1.**
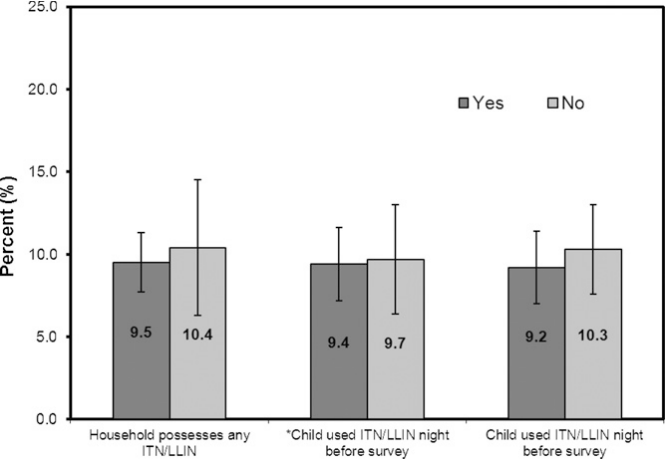
Malaria parasite infection prevalence among children < 5 years old by ITN ownership and use in Luangwa District, Zambia, in 2008 and 2010. *Among ITN-owning households. χ^2^ tests showed no significant differences in malaria parasite infection prevalence between ITN household possession or use variables.

At the end of the malaria transmission season, the malaria parasite infection prevalence was 7.0% (95% CI = 5.0–9.0%) among children < 5 years old in 2008 and 12.6% (95% CI = 9.9–15.3%) in 2010. The overall parasite infection prevalence was 9.7% (95% CI = 8.0–11.4%) across both years. Bivariate analyses showed no difference in parasite prevalence between children in households with or without an ITN or between children who used an ITN or did not use an ITN the previous night (Figure 1).

**Figure 2.**
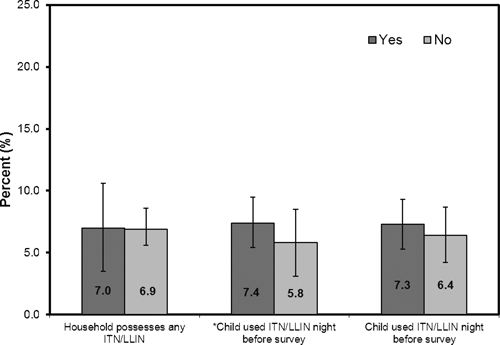
Severe anemia prevalence among children 6–59 months old by ITN ownership and use in Luangwa District, Zambia, in 2008 and 2010. *Among ITN-owning households. χ^2^ tests showed no significant differences in severe anemia prevalence between ITN household possession or use variables.

At the end of the malaria transmission season, the prevalence of severe anemia among children 6–59 months was 5.8% (95% CI = 3.8–7.9%) in 2008, 8.0% (95% CI = 5.7–10.3%) in 2010, and 6.9% (95% CI = 5.4–8.5%) over both years. Bivariate analyses showed no difference in severe anemia between children in households with or without an ITN or between those that used or did not use an ITN the previous night (Figure 2).

There was a strong positive correlation between the prevalence of malaria parasite infection and severe anemia (χ^2^ = 73.9; *P* < 0.001) across both years. Overall, 38.5% of children with severe anemia also had a malaria parasite infection, whereas 26.3% of children with a malaria parasite infection also had severe anemia.

Results from the four logistic regressions (two per outcome of parasite prevalence and severe anemia prevalence), controlling for child age and sex, location of household in the district, survey year, and household wealth, suggest that neither ITN possession nor ITN use was significantly associated with malaria parasite infection prevalence or severe anemia in children in this context (Tables 2 and 3). Children living in the northern part of the district were two times as likely to have a malaria parasite infection compared with their counterparts in the southern part of the district across all models. Similarly, children living in the northern part of the district were also two times as likely to have severe anemia across all models.

Additional regressions were performed using ITN to occupant ratio as a second test variable and proxy for ITN ownership and whether anyone in the house slept under an ITN as a test variable and proxy for ITN use (results not shown); results of these additional regressions were similar to the results that we present here and also showed that ITN possession and use were not significantly associated with malaria parasite infection prevalence or severe anemia in this context.

## Discussion

Results from the 2008 and 2010 household surveys show that Luangwa District, Zambia, has achieved near-universal coverage of ITNs, with > 80% of households possessing ≥ 1 ITN. The majority of children in the sample (58.4%) lived in households with adequate intra-household access to an ITN, defined as ≥ 1 ITN for every two household occupants. ITN use among those in ITN-owning households increased from just over one-half (52.8%) in 2008 to over three-quarters (85.5%) in 2010.

At the end of the malaria transmission seasons in 2008 and 2010, the prevalence of children with a malaria parasite infection in Luangwa District was only 9.7%, whereas the prevalence of severe anemia among these children aged 6–59 months was 6.9%.

Results of the 2010 Zambia National MIS are not yet available. However, the prevalence of malaria parasite infection in Luangwa in 2008 (7.0%), with near-universal coverage of ITNs, was almost one-half that of the rest of rural Zambia during the malaria transmission season in 2008, which had a parasite infection prevalence of 12.4% and ITN household possession of 63.9%.^9^ This is also one-quarter of the malaria parasite prevalence observed across rural Zambia in 2006 just after the malaria transmission season (28.8%), where the level of households possessing ITNs was only 44.2%.^10^ The prevalence of severe anemia in 2008 (5.8%) was similar to that in the rest of rural Zambia during this time in 2008, which had a prevalence of 4.3%, but was only one-quarter of the prevalence in 2006 of 15.4% across rural Zambia.^9^,^10^

Although one might expect that the association of severe anemia in children with a malaria parasite infection to decrease in such an area of near-universal coverage of ITNs, there remains a strong association among these conditions within this area of intervention-suppressed transmission (χ^2^ = 73.9; *P* < 0.001). This suggests that, even with high ITN coverage in areas of once stable *P. falciparum* transmission, chronic malaria parasite infections remain a significant risk factor for severe anemia in children 6–59 months.

Within this context of intervention-suppressed transmission with high ITN coverage, our data were unable to detect a significant difference in malaria parasite infection prevalence or severe anemia among children living in households with or without an ITN. Although not statistically significant, we found a 5% (95% CI = −60–44%) reduction in malaria parasite prevalence among children in ITN-possessing households compared with those in households without an ITN. This is less than one-third of the 17% reduction detected by the trials, which compared children in villages with and without ITNs.^1^ We identified only one trial that assessed the association of household ITN possession with malaria parasite infection prevalence in children. It showed ITNs to be associated with a 50% reduction in malaria parasite infection prevalence within an area with about 35% household ITN coverage.^11^,^12^

Our analyses were also unable to detect a significant protective effect of sleeping under an ITN the previous night against malaria parasite infection or severe anemia prevalence among all children (29%; 95% CI = −10–53%) and among those within ITN-owning households (28%; 95% CI: −21–57%). We identified four studies that published data on the association between ITN use and malaria parasite infection prevalence under program conditions. Reductions in malaria parasite infection prevalence among children who slept versus did not sleep under ITNs ranged from 39% to 63%; all were significant.^13^–^16^ All of these studies reported less than 50% household ITN coverage.

Within this area of near-universal ITN coverage, we hypothesize that our null results are explained in part by the community effect of ITNs, where children unprotected by ITNs benefited from an overall suppression of transmission.^4^,^5^,^17^ Although our data do not provide definitive evidence of a community effect, our results do suggest that at least partial protection has been conferred on the population in Luangwa District, as evidenced by similar levels of malaria parasite infection prevalence and severe anemia prevalence, irrespective of ITN household possession or use.

This study has several important limitations. First, this study was slightly underpowered to detect such small differences in the prevalence of malaria parasite infection and severe anemia by ITN possession and use. Second, although there is anecdotal evidence that malaria transmission was once high within this area of Zambia, we could not identify published data that quantified the level of malaria transmission before the ITN scale-up in 2005; thus, an assessment of the relative decline in malaria transmission associated with the ITN scale-up was precluded. Lastly, we did not collect parasite or hemoglobin data from the population older than 4 years and therefore, were unable to assess the relationship between ITNs and malaria parasite infections among this population.

Our results suggest that, with near-universal coverage of ITNs, malaria parasite infection prevalence remains at 10% in this area of Zambia. Such prevalence represents a substantial pool of parasites that will sustain transmission. In addition to maintaining universal ITN coverage, it will be essential for the malaria control program to achieve high ITN use and laboratory diagnosis and treatment of all fevers among all age groups to further reduce the malaria burden in this area.

## Figures and Tables

**Table 1:** ITN ownership and use among children tested for malaria parasites in Luangwa District, Zambia, in 2008 and 2010

	Child lives in household owning ³ 1 ITN (*N* = 1,190)		Child lives in household with ITN to occupant ratio ≥ 1:2 (*N* = 1,190)		Child used ITN previous night* (*N* = 969)		Child lives in household with ITN used by anyone previous night* (*N* = 969)	
	%	95% CI	%	95% CI	%	95% CI	%	95% CI
Age								
0–11 months	78.9	73.4–84.3	16.8	12.0–21.6	65.6	58.5–72.6	68.9	62.0–75.7
12–23 months	79.3	74.1–84.5	21.5	16.4–26.6	70.4	64.1–76.6	71.9	65.7–78.0
24–35 months	82.3	77.5–87.0	19.8	14.6–24.9	76.0†	70.0–81.9	77.5†	71.6–83.3
36–47 months	80.9	75.6–86.1	17.8	12.8–23.0	65.9	59.1–72.8	67.0	60.2–73.8
48–59 months	85.9*	81.3–90.5	19.2	14.1–24.4	62.0	55.0–68.4	62.7	56.0–69.3
Sex								
Male	81.9	78.4–85.4	18.6	15.2–22.0	66.4	61.8–71.0	68.2	63.7–72.7
Female	81.0	77.2–84.7	19.6	15.9–23.2	69.7	65.3–74.1	71.2	66.9–75.5
North Luangwa (≥ 50 km from Boma)	83.4	76.0–83.7	18.7	15.3–23.9	68.4	63.1–73.7	70.4	65.4–75.5
South Luangwa (< 50 km from Boma)	79.8	79.3–87.6	19.6	15.0–22.3	67.7	63.0–72.3	69.0	64.4–73.6
Household Wealth								
Poorest	72.3	65.4–79.1	12.0	7.3–16.8	70.7	63.1–78.3	72.2	64.8–79.7
Poor	81.8†	75.5–88.1	16.0	10.0–21.9	66.8	59.3–74.3	66.8	59.3–74.3
Middle	80.2	73.6–86.7	20.3†	13.9–26.6	66.7	58.6–74.7	68.3	60.3–76.3
Rich	85.3‡	79.7–90.9	19.3	12.8–25.7	65.6	56.9–74.3	69.4	61.2–77.5
Richest	90.9‡	86.1–95.6	31.5‡	23.6–39.4	70.4	62.5–78.2	72.1	64.3–79.8
Total	81.4	78.5–84.3	19.1	16.3–21.8	68.0	64.6–71.4	69.7	66.4–73.0

* Among children living in households owning an ITN.

† *P* < 0.05.

‡ *P* < 0.01.

**Table 2:** Logistic regressions predicting odds of parasite infection and severe anemia relative to ITN ownership among children providing a blood sample in Luangwa District, Zambia, in 2008 and 2010

	Malaria parasite prevalence (*N* = 1,190)		Severe anemia: Hb < 8 g/dL (*N* = 1,125)*	
	AOR	95% CI	AOR	95% CI
Household possesses any ITN	0.95	0.56–1.60	1.04	0.56–1.95
Age				
0–11 months (reference)	1.00		1.00	
12–23 months	2.75†	1.18–6.40	1.13	0.56–2.26
24–35 months	3.39†	1.46–7.87	0.99	0.48–2.05
36–47 months	3.52†	1.51–8.18	0.37†	0.15–0.90
48–59 months	3.82†	1.73–8.45	0.48	0.21–1.09
Sex				
Male (reference)	1.00		1.00	
Female	0.67	0.44–1.03	0.57†	0.35–0.93
South Luangwa (< 50 km from Boma; reference)	1.00		1.00	
North Luangwa (≥ 50 km from Boma)	2.21‡	1.43–3.41	1.93‡	1.15–3.22
Household wealth				
Poorest (reference)	1.00		1.00	
Poor	0.92	0.52–1.64	0.51	0.24–1.10
Middle	0.80	0.44–1.43	0.82	0.41–1.64
Rich	0.77	0.42–1.41	1.57	0.81–3.05
Richest	0.29†	0.11–0.73	0.44	0.17–1.14
Year				
2008 (reference)	1.00		1.00	
2010	1.36†	1.10–1.68	1.16	0.92–1.48
Design degrees of freedom	769		750	
*F*(12,759)	3.97			
*F*(12,739)			3.37	
Probability > *F*	0.0000		0.0001	

AOR = adjusted odds ratio; CI = confidence interval.

* Ages 6–59 months old.

† *P* < 0.05.

‡ *P* < 0.01.

**Table 3:** Logistic regressions predicting odds of parasite infection and severe anemia relative to ITN use among children providing a blood sample and living within an ITN-owning house in Luangwa District, Zambia, in 2008 and 2010

	Malaria parasite prevalence (*N* = 969)		Severe anemia: Hb < 8 g/dL (*N* = 925)*	
	OR	95% CI	OR	95% CI
Child used ITN night before the survey	0.72	0.43–1.21	1.19	0.60–2.37
Age				
0–11 months (reference)	1.00		1.00	
12–23 months	2.46†	1.02–5.96	1.29	0.60–2.80
24–35 months	2.57†	1.06–6.29	1.08	0.48–2.43
36–47 months	2.60†	1.09–6.19	0.42	0.16–1.09
48–59 months	2.88†	1.28–6.45	0.49	0.20–1.21
Sex				
Male (reference)	1.00		1.00	
Female	0.71	0.45–1.13	0.51†	0.30–0.87
South Luangwa (< 50 km from Boma; reference)	1.00		1.00	
North Luangwa (≥ 50 km from Boma)	2.15‡	1.35–3.41	2.03†	1.14–3.60
Household wealth				
Poorest (reference)	1.00		1.00	
Poor	0.83	0.44–1.57	0.53	0.22–1.26
Middle	0.71	0.37–1.39	0.82	0.37–1.83
Rich	0.80	0.41–1.53	1.79	0.85–3.76
Richest	0.31†	0.12–0.80	0.41	0.14–1.21
Year				
2008 (reference)	1.00			
2010	1.49†	1.16–1.92	1.11	0.82–1.49
Design degrees of freedom	629		614	
*F*(12,618)	3.13		3.27	
*F*(12,603)	0.0002		0.0001	
Probability > *F*				

AOR = adjusted odds ratio; CI = confidence interval.

* Ages 6–59 months old.

† *P* < 0.05.

‡ *P* < 0.01.
